# WHO recommendations for resilient health systems

**DOI:** 10.2471/BLT.22.287843

**Published:** 2022-04-01

**Authors:** Tedros Adhanom Ghebreyesus, Zsuzsanna Jakab, Michael J Ryan, Jaouad Mahjour, Suraya Dalil, Stella Chungong, Gerard Schmets, Geraldine Mcdarby, Redda Seifeldin, Sohel Saikat

**Affiliations:** a World Health Organization, Geneva, Switzerland.; b Health Emergencies Programme, World Health Organization, Geneva, Switzerland.; c Universal Health Coverage and Life-course Division, World Health Organization, Avenue Appia 20, 1211 Geneva 27, Switzerland.; d Health Security Preparedness Department, World Health Organization, Geneva, Switzerland.

On 19 October 2021 the World Health Organization (WHO) launched its position paper *Building health systems resilience for universal health coverage and health security during the COVID-19 pandemic and beyond*^1^ to support countries in recovering from the pandemic. The launch took place amid unprecedented public and political calls for greater resilience in health systems. 

Despite our collective experience with public health emergencies, the coronavirus disease 2019 (COVID-19) has demonstrated that national health systems were poorly prepared for a pandemic of this scale. While examples exist of good practice and innovation that kept mortality or case counts relatively low, this apparent success often came at the expense of economic activity, mental health or personal freedoms.^2^ Globally, deficiencies in coordination, transparency and timeliness of data sharing prevented us from moving quickly, learning from each other and supporting each other. The current and protracted lack of equity in access to vaccines^3^ demonstrates the need for global preparedness, including the development of pre-crisis international agreements on access to innovation.

COVID-19 has exposed the weaknesses in health, economic and social systems worldwide, with countries experiencing significant disruptions and massive economic losses due to the pandemic and response efforts.^4^ No country is unaffected, and wealth did little to insulate countries against the negative effects of the pandemic. As with previous public health emergencies, the indirect deaths associated with health and social service disruptions and economic breakdown may surpass those directly caused by the virus.^5^^,^^6^ As always, the brunt is borne by the most vulnerable populations, including those in countries under protracted conflict.^7^ The pandemic has demonstrated that health is the foundation of socioeconomic development and that we are only as safe as the most vulnerable among us. We have seen that when health is at risk, all other sectors are at risk.^8^ Health systems are a vital first line of defence, not only against pandemics but against the physical and mental health issues that prevent us from reaching our full potential, both individually and collectively.

Unfortunately, at the beginning of the COVID-19 outbreak, much of what was learned from past experiences with the severe acute respiratory syndrome, Middle East respiratory syndrome or Ebola virus and other public health emergencies had not been applied in many health systems. Many countries did not prioritize health emergency preparedness, with many lacking the capacities required under the International Health Regulations (2005).^9^^,^^10^ Fragmented approaches to policy, planning, programming, implementation, and monitoring and evaluation continue to cause inefficient use of resources and perpetuate critical foundational gaps in health systems. Health investments have often been misaligned with needs, with prioritization of individual health care over public health interventions, emergency response often superseding preparedness, prevention and promotion, and with little emphasis on primary health care or on communities as the centre of decision-making.^11^^,^^12^

Issues beyond the health sector such as changing demographic patterns, climate change, changing land use, deforestation and increased animal-human proximity, coupled with increasing population density and globalization are increasing the likelihood of further pandemics or other crises. Now is the time for all sectors to work together on health. The delivery of COVID-19 vaccines in under a year as opposed to the usual 10–15 years demonstrates the astounding progress that is possible when attention and resources are focused on a common task.

Therefore, still amidst this pandemic and its economic, social and health consequences, we have the duty to do things differently. The only choice is to invest, making smarter and more intelligent use of all our resources, to create fairer and more resilient health systems that will be able to prevent and prepare for future pandemics. Doing so will demonstrate that we have learnt the lesson of this pandemic – that health is not a cost to be contained, but an investment to be nurtured. Given the massive return in terms of avoiding future economic and social losses, investing in resilient health systems that provide high-quality essential health services should not be considered a luxury anymore, but as the foundation of social, economic and political stability.

WHO calls on leaders and policy-makers within health, finance and other sectors to act on the seven recommendations of the position paper on building health systems resilience to: (i) leverage the current response to strengthen both pandemic preparedness and health systems; (ii) invest in essential public health functions including those needed for all-hazards emergency risk management; (iii) build a strong primary health-care foundation; (iv) invest in institutionalized mechanisms for whole-of-society engagement; (v) create and promote enabling environments for research, innovation and learning; (vi) increase domestic and global investment in health system foundations and all-hazards emergency risk management; and (vii) address pre-existing inequities and the disproportionate impact of COVID-19 on marginalized and vulnerable populations.^1^

## References

[1] Building health systems resilience for universal health coverage and health security during the COVID-19 pandemic and beyond: WHO position paper. Geneva: World Health Organization; 2021. Available from: http://apps.who.int/iris/handle/10665/346515 [cited 2021 Nov 10].

[2] Shared responsibility, global solidarity: responding to the socio-economic impacts of Covid-19. New York: United Nations; 2020. Available from: https://unsdg.un.org/sites/default/files/2020-03/SG-Report-Socio-Economic-Impact-of-Covid19.pdf [cited 2022 Mar 1].

[3] Vaccine inequity undermining global economic recovery. Geneva: World Health Organization; 2021. Available from: https://www.who.int/news/item/22-07-2021-vaccine-inequity-undermining-global-economic-recovery [cited 2022 Mar 1].

[4] A UN framework for the immediate socio-economic response to COVID-19. New York: United Nations; 2020. Available from: https://unsdg.un.org/sites/default/files/2020-04/UN-framework-for-the-immediate-socio-economic-response-to-COVID-19.pdf [cited 2021 Dec 3].

[5] Roberton T, Carter ED, Chou VB, Stegmuller AR, Jackson BD, Tam Y, et al. Early estimates of the indirect effects of the COVID-19 pandemic on maternal and child mortality in low-income and middle-income countries: a modelling study. Lancet Glob Health. 2020 Jul;8(7):e901–8. 10.1016/S2214-109X(20)30229-1 32405459 PMC7217645

[6] Tracking covid-19 excess deaths across countries [internet]. The Economist. 2022. Available from: https://www.economist.com/graphic-detail/coronavirus-excess-deaths-tracker [cited 2022 Mar 1].

[7] Covid-19 and fragile settings, Policy Brief. UHC Partnership 2030; 2020. Available from: https://www.uhc2030.org/fileadmin/uploads/uhc2030/Documents/About_UHC2030/UHC2030_Working_Groups/2017_Fragility_working_groups_docs/UHC2030_Policy_brief_COVID19_and_fragile_settings_WEB1.pdf [cited 2022 March 1].

[8] Building health systems resilience for universal health coverage and health security during the COVID-19 pandemic and beyond: WHO position brief. Geneva: World Health Organization; 2021. Available from:https://www.who.int/publications/i/item/WHO-UHL-PHC-SP-2021.02 [cited 2021 Nov 10].

[9] The Global Health Observatory. Geneva: World Health Organization; 2022. Available from: https://www.who.int/data/gho/data/major-themes/health-emergencies [cited 2021 Nov 18].

[10] A review of joint external evaluations and national action plans for health security in 13 countries from a health systems perspective. Geneva: World Health Organization; 2021. Available from: https://www.who.int/publications/i/item/9789240033276 [cited 2021 Dec 3].

[11] 21st century health challenges – can the essential public health functions (EPHFs) make a difference? Geneva: World Health Organization; 2021. Available from: https://www.who.int/publications/i/item/9789240038929 [cited 2022 Mar 1].

[12] Fostering resilience through integrated health system strengthening: technical meeting report. Geneva: World Health Organization; 2021. Available from: https://www.who.int/publications/i/item/9789240033313 [cited 2021 Dec 3].

